# An examination of resting-state functional connectivity in patients with active Crohn’s disease

**DOI:** 10.3389/fnins.2023.1265815

**Published:** 2023-12-06

**Authors:** Gita Thapaliya, Sally Eldeghaidy, Shellie J. Radford, Susan T. Francis, Gordon William Moran

**Affiliations:** ^1^Division of Child and Adolescent Psychiatry, Department of Psychiatry and Behavioral Sciences, Johns Hopkins University School of Medicine, Baltimore, MD, United States; ^2^Division of Food, Nutrition and Dietetics, School of Biosciences, The University of Nottingham, Loughborough, United Kingdom; ^3^Sir Peter Mansfield Imaging Centre, School of Physics and Astronomy, The University of Nottingham, Nottingham, United Kingdom; ^4^NIHR Nottingham Biomedical Research Centre, Nottingham University Hospitals NHS Trust and School of Medicine, The University of Nottingham, Nottingham, United Kingdom; ^5^Translational Medical Sciences Unit, University of Nottingham, Nottingham, United Kingdom

**Keywords:** Crohn’s disease, gut-brain axis, gut inflammation, systemic inflammation, functional connectivity

## Abstract

**Background:**

Alterations in resting state functional connectivity (rs-FC) in Crohn’s Disease (CD) have been documented in default mode network (DMN) and frontal parietal network (FPN) areas, visual, cerebellar, salience and attention resting-state-networks (RSNs), constituting a CD specific neural phenotype. To date, most studies are in patients in remission, with limited studies in active disease.

**Methods:**

Twenty five active CD cases and 25 age-, BMI- and gender-matched healthy controls (HC) were recruited to a resting-state-functional Magnetic Resonance Imaging (rs-fMRI) study. Active disease was defined as C-reactive protein>5 mg/dL, faecal calprotectin>250 μg/g, or through ileocolonoscopy or MRE. rs-fMRI data were analysed using independent component analysis (ICA) and dual regression. Differences in RSNs between HCs and active CD were assessed, and rs-FC was associated with disease duration and abdominal pain.

**Results:**

Increased connectivity in the FPN (fusiform gyrus, thalamus, caudate, posterior cingulate cortex, postcentral gyrus) and visual RSN (orbital frontal cortex) were observed in CD versus HC. Decreased activity was observed in the salience network (cerebellum, postcentral gyrus), DMN (parahippocampal gyrus, cerebellum), and cerebellar network (occipital fusiform gyrus, cerebellum) in CD versus HCs. Greater abdominal pain scores were associated with lower connectivity in the precuneus (visual network) and parietal operculum (salience network), and higher connectivity in the cerebellum (frontal network). Greater disease duration was associated with greater connectivity in the middle temporal gyrus and planum temporale (visual network).

**Conclusion:**

Alterations in rs-FC in active CD in RSNs implicated in cognition, attention, emotion, and pain may represent neural correlates of chronic systemic inflammation, abdominal pain, disease duration, and severity.

## 1 Introduction

### 1.1 Structural and functional brain alterations in Crohn’s disease

Crohn’s disease (CD) is a chronic inflammatory bowel disease (IBD) characterized by a chronic inflammatory response, with symptoms ranging from fatigue, abdominal pain, diarrhea, psychological comorbidities, and extraintestinal manifestations (EIMs) (Lamb et al., 2019). Consistent exposure to pain and inflammation in CD may impact the functional architecture of the brain. A growing body of literature has demonstrated that CD and its comorbidities is associated with a distinct intrinsic neural phenotype attributed to brain structural and functional alterations (Kong et al., 2021; Yeung, 2021). Systemic inflammation, chronic abdominal pain, dysregulated gut-brain signaling pathways, and symptom-specific mechanisms may be involved in such brain structural and functional alterations. Structural changes of the brain in active CD have previously been demonstrated by our group in relation to symptoms such as fatigue, abdominal pain and EIMs (Thapaliya et al., 2023), with others demonstrating structural changes in remission (Yeung, 2021). To explore brain behavior associations in CD, task-based fMRI studies to stress (Agostini et al., 2013, 2017) and pain (Rubio et al., 2016) have revealed differential brain responses in CD patients relative to healthy controls, indicating an atypical response to unpleasant stimuli.

### 1.2 The importance of studying functional connectivity patterns in Crohn’s disease

Resting state fMRI (rs-fMRI) measures spontaneous brain activity at rest. This does not depend on how a patient performs to a task, meaning it is less burdensome to the patient. rs-fMRI helps to understand how different brain regions communicate with each other to generate functional connectivity patterns. A resting state network (RSN) constitutes a group of brain regions that are spatially distant, but functionally connected and continuously communicating (Smitha et al., 2017). Comparisons of functional connectivity between CD patients during active disease and remission, and with healthy controls, may aid the identification of neural biomarkers or signatures, and changes in connectivity patterns to therapeutic interventions guiding personalized treatment approaches.

### 1.3 Functional connectivity alterations reported in Crohn’s disease in remission

Alterations in a number of RSNs, namely the default mode network (DMN), frontal–parietal network (FPN) and executive control network (ECN), frontoparietal network (FPN), salience network (SN), dorsal attention network (DAN), sensory-motor network (SMN), cerebellar network and visual networks have been implicated in CD. The functions of these various RSNs are described in detail in Supplementary Table S1. A recent meta-analysis (Yeung, 2021) in CD reported reduced resting state functional connectivity (rs-FC) in the cingulate gyrus, which is an integral part of the DMN. Consistent with this meta-analysis, recent studies have reported reduced neural synchronization in key hubs of the DMN (Kornelsen et al., 2020; Thomann et al., 2021; Zhang et al., 2022) in association with greater abdominal pain (Chen et al., 2023). In contrast, another study reported increased rs-FC in the DMN in CD in remission between the right precuneus and right posterior cingulate cortex (PCC) as compared with HCs (Hou et al., 2019). Apart from the DMN, increased rs-FC in the ECN has been reported between the right middle frontal gyrus and the right inferior parietal lobe (Hou et al., 2019). Further, increased rs-FC between the FPN and salience network (SN) (Kornelsen et al., 2020), and greater rs-FC in several regions in the cerebellar, visual, and SN has been found in a mixed CD group compared with HCs. Finally, reduced rs-FC in several regions of the right FPN and dorsal attention network (DAN) has also been reported in CD in remission (Mallio et al., 2020).

### 1.4 Limited functional connectivity studies in active Crohn’s disease

Notably, the majority of the rs-FC studies conducted thus far have been conducted in CD participants who are in remission, and until recently no published studies included participants in the active disease phase (Huang et al., 2022; Kong et al., 2022; Li et al., 2022; Prüß et al., 2022; Agostini et al., 2023; Goodyear et al., 2023). Of these studies in the active phase, one study restricted their analysis to the anterior cingulate cortex ACC (part of the DMN) and showed rs-FC as well as structural and metabolic changes in active CD (Kong et al., 2022). Decreased rs-FC in the left calcarine of the primary visual network (Li et al., 2022) was found in active CD participants, with these changes associated with greater CD duration. Greater rs-FC in the somatosensory cortex in the SN in active IBD patients with chronic pain was found regardless of their inflammatory status relative to HCs (Prüß et al., 2022). Most recently, increased rs-FC has been reported within the left FPN (in the superior parietal lobe) in CD in remission relative to active CD, as well as decreased rs-FC in the motor network (in parietal and motor regions in active CD relative to HC; Agostini et al., 2023). These mixed results highlight the need for more studies in CD patients with active disease, as well as prospective studies to track connectivity patterns in RSNs in active disease and remission and changes related to improvement in symptoms.

### 1.5 Study aims and objectives

We present results of an exploratory study with the primary aim being to study alterations in rs-FC in active CD, to expand upon the currently limited literature.

Our primary objective is to compare rs-FC in active CD to HCs. Our secondary objective is to identify neural correlates of abdominal pain, fatigue and disease duration in active CD participants.

## 2 Methods

### 2.1 Basic protocol and patient recruitment

This case–control study received research ethics committee approval from the National Research Ethics Service (NRES) Committee East Midlands (REC reference 14/EM/0192 on 10/07/2015) and the protocol was registered with clinicaltrials.gov (NCT02772458). CD participants were identified through a clinical database search and expression of interest list, and recruited from the IBD Clinic at Nottingham University Hospitals. HCs were recruited from a participant database in the National Institute of Health Research (NIHR) Nottingham Biomedical Research Centre, and local healthy populations of Nottingham University Hospitals and the University of Nottingham recruited through study fliers and social media. All CD participants and HCs read a participant information sheet and gave their written informed consent before recruitment to the study.

CD participants’ disease activity was defined through one or more objective markers of inflammation defined as faecal calprotectin (FCP) of >250 μg/g or C-reactive protein (CRP) > 5 g/dL, or through recent ileocolonoscopy (defined as presence of ulcerations), CT, magnetic resonance enterography (MRE) showing active inflammatory disease (defined as presence of post-contrast enhancement or visible mucosal ulcerations on cross-sectional imaging). This active disease characterization is supported by the European Crohn’s and Colitis organization (see review Maaser et al., 2019) CD clinical symptoms were measured at inclusion using the Harvey-Bradshaw Index (HBI) score, a simple index based on 5 items assessing symptoms and complications (including general well-being, abdominal pain, number of liquid or soft stools per day, abdominal mass and EIM) (Harvey, 1980). Exclusion criteria included malignant disease, BMI <18 or 35 kg/m^2^, significant cardiovascular or respiratory disease, diabetes mellitus, current infection, neurological or cognitive impairment, significant physical disability, significant hepatic disease or renal failure, abnormal blood results other than those explained by CD including bleeding diatheses (in the case of HCs all unexplained blood results were exclusion criteria), pregnancy or breastfeeding, recent corticosteroid exposure (in the past 3 months), severe CD where a delay in a change in medical treatment for 1–2 weeks would not be clinically advisable, or contraindication to MRI (e.g., pacemaker). Stable doses of immunosuppressive agents or biological agents were permitted details of these are provided in Supplementary Table S2.

### 2.2 Clinical assessments

The IBD-Fatigue self-assessment scale (Section 1, which comprises 5 questions assessing frequency and severity of fatigue which has been validated and shown to have excellent test–retest stability in IBD) was used to identify IBD specific fatigue and its severity, frequency, and duration, where 0 = no fatigue, 1–10 = moderate fatigue, 11–20 = severe fatigue (Czuber-Dochan et al., 2014). Abdominal pain was assessed using a 100 mm VAS (Mujagic et al., 2015) with CD patients asked “Do you currently suffer from abdominal (tummy) pain?” yes/no, if yes “How severe is your abdominal (tummy) pain?” on a scale from 0 to 100. It should be noted that this is the level of subjective pain of the CD patients experienced at the time of their MRI scan despite some being on medications. The presence or absence of EIM was based on CD participants’ responses to the “complications” section of the HBI, where CD patients were asked to check boxes that apply (i) none (ii) arthralgia, (iii) uveitis, (iv) erythema nodosum, (v) aphthous ulcers, (vi) pyoderma gangrenosum, (vii) anal fissure, (viii) new fistula, and (ix) abscess. Serum levels of interleukin-6 (IL-6), interleukin-1 beta (IL-1 β), and tumour necrosis factor (TNFα) were measured using an immunoassay kit (Duoset ELISA Development, R&D Systems, Inc., United States) as previously described (Negm et al., 2019).

Depression and anxiety symptoms were measured using the Hospital Anxiety and Depression Scale (HADS) (Zigmond and Snaith, 1983), a 14-item questionnaire graded on a 4-point Likert scale with subscales of anxiety and depression, with a sum score ranging from 0 to 21 for each and a cut-off value of >7 on either of the 2 subscales. Scores of 0–7 are considered normal, 8–10 are indicative of mild anxiety/depression symptoms, 11–14 are indicative of moderate anxiety/depression symptoms, and 15–21 are indicative of severe anxiety/depression symptoms (Mikocka-Walus et al., 2016).

### 2.3 Data acquisition

After an overnight fast, participants underwent a 10-min resting-state BOLD fMRI scan on a 3 T Achieva scanner (Philips Medical Systems, Best, Netherlands). Images were collected with a 32-channel receive head coil using a double-echo gradient-echo EPI (GE-EPI) acquisition scheme (parameters: echo time (TE) of 20/49 ms, 64 × 64 matrix, 3 mm isotropic voxels, 44 axial slices, TR = 2 s). Subjects were instructed to lay still inside the scanner with their eyes open. Physiological heart rate and respiratory data were collected throughout. A 3D T_1_-weighted MPRAGE image (1 mm isotropic resolution, TE/TR = 8.3/3.8 ms, flip angle = 8°, SENSE factor = 2,160 slices, 256 × 256 matrix) was also acquired.

#### 2.3.1 Pre-processing

Specific nuisance signals were regressed out during the pre-processing steps. First, physiological noise signal components associated with cardiac pulsation and respiration was removed using RETROICOR (Glover et al., 2000). Pre-processing and analyses were then performed using the FMRIB Software Library (FSL).^1^ Head motion was corrected using multi-resolution rigid body co-registration of volumes using MCFLIRT (Jenkinson et al., 2002) and the six motion parameters (3 translational and 3 rotational regressors) were regressed out from the data. In addition, the mean time courses of cerebral white matter, ventricles, and whole brain were used as signal regressors and masking of non-brain voxels was performed. Brain extraction was then performed on the motion-corrected fMRI volumes and MPRAGE datasets using the Brain Extraction Tool software. fMRI volumes were then registered to their subject-specific MPRAGE and moved to MNI152 standard space by applying the transform of the co-registration of the MPRAGE volume to MNI152 standard space. fMRI images were smoothed with a 6-mm spatial filter and a high-pass temporal filter cut-off of 100 s applied.

#### 2.3.2 ICA analysis

The Multivariate Exploratory Linear Decomposition into Independent Components (MELODIC) tool was used to perform single-session independent component analysis (ICA) on all subjects to produce 30 component maps. Any nuisance components arising from noise and motion-related artifacts were removed following visual inspection (Griffanti et al., 2017) and the *fslregfilt* function was used to regress out nuisance components from each dataset. We chose to estimate 30 component maps, which is a typical number used in the literature, as this has been shown to provide a good trade-off between providing a good representative of the fMRI data structure whilst making the analysis and interpretation more manageable (Wang and Li, 2015; Vergun et al., 2016). Group ICA was then performed on the noise-free datasets from the single session ICA step using multisession temporal concatenation to produce 30 component maps representing the average RSNs of the entire study population comprising CD participants and HCs, as well as a group ICA performed for the CD group only and the HC group only.

#### 2.3.3 Group ICA with dual regression

Dual regression was performed using the HC group 30 component maps as the network template. The HC group network was chosen as this is more robust in the absence of disease-related alterations and more sensitive in detecting group differences in the dual regression as compared with using the entire (CD and HC group) study population component maps (Rytty et al., 2013). Variance normalization was used, reflecting the differences in both activity and spatial spread of RSNs.

Dual regression was carried out in three stages. In Stage 1, the subject-specific time course for each network template was extracted using a multivariate spatial regression of the template maps against each subject’s fMRI data. In Stage 2, subject-specific time courses from Stage 1 were used in a second multivariate temporal regression against the subjects’ fMRI data to identify subject-specific spatial maps corresponding to each network template of interest. In Stage 3, a two-sample unpaired t-test between the CD and HC group was performed, where different component maps were collected across subjects into single 4D files (i.e., per original ICA map) and tested voxel-wise by non-parametric permutation using the FSL randomize tool (Winkler et al., 2014) with 5,000 permutations and TFCE to control for multiple comparisons. Differences between groups were tested at *p* < 0.05 with voxel-wise changes in the FSL randomize tool. It is important to highlight that using the spatial maps output by dual-regression in such a test can result in areas of significant difference both “within” and not “within” the group-average HC component map for that RSN. Areas not “within” the group-average HC component map for that RSN can be interpreted to represent that the connectivity of this area of the RSN is different between the two groups. For example, this can result if an area has a weak positive synchrony, with the main areas of a RSN in the HC group and a weak negative synchrony in the CD group.

For the CD group, at stage 2 a General linear model (GLM) was created to investigate the correlation between the RSNs and clinical scores of disease duration, abdominal pain score, and IBD fatigue score, as well as anxiety and depression, using permutation-based non-parametric testing (5,000 permutations) and TFCE, with cluster significance threshold of *p* < 0.05. We then tested if these survived multiple comparisons using Bonferroni method. The Juelich histological atlas incorporated in FSL and the Harvard-Oxford cortical and subcortical atlases (Harvard Centre for Morphometric Analysis) provided within the FSL4 software were used to identify anatomical characteristics of the resulting maps.

### 2.4 Analysis of clinical and behavioral data

Analyses of non-imaging data were carried out using SPSS Statistics version 28.0. All variables were tested for normality using the Shapiro–Wilk test. Normal data were expressed as mean ± standard error of the mean (SEM) and non-parametric data as median (interquartile range, IQR). Correlation between different variables was evaluated using the Spearman for non-parametric data and Pearson’s correlation for parametric data.

## 3 Results

**Figure 1:**
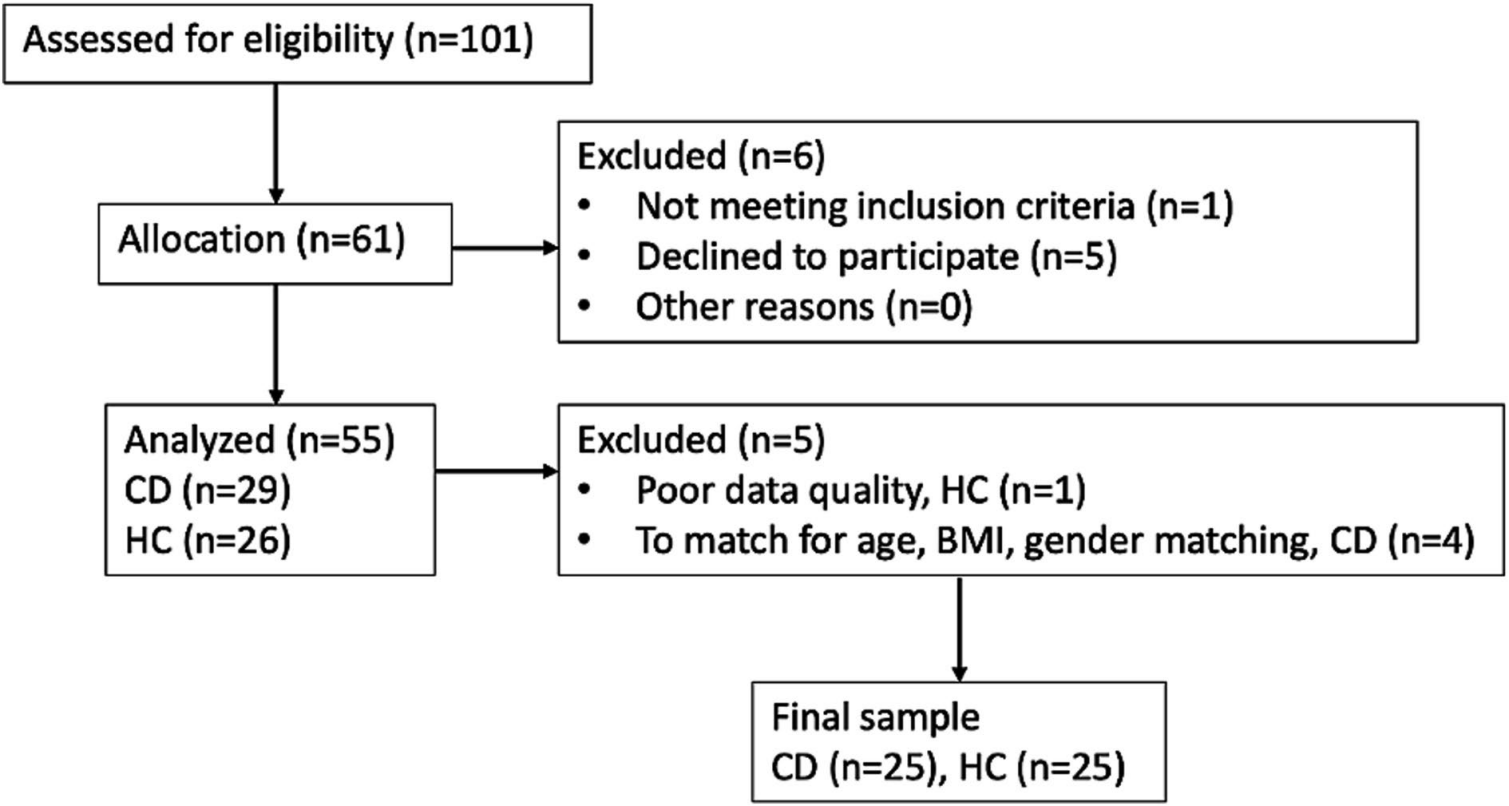
Consort diagram outlining the number of CD participants and HCs included in the study.

In total, 25 CD participants and 25 age, BMI, and gender-matched HCs were included in this study. Figure 1 provides a consort diagram outlining the number of participants recruited for the study. Table 1 provides the demographic, behavioral, and clinical characteristics of the CD participants and HCs. In 20/25 cases, disease activity was defined through ileocolonoscopy or MRE and in 4/5 cases it was defined through CRP and fecal calprotectin together. Further information on each of the CD patient characteristics including the measure(s) used to define active disease and any medications are provided in Supplementary Table S2. The CD and HC groups did not differ in any of their behavioral characteristics, except for the HADS depression score, for which CD participants had significantly greater scores than HCs (*p* = 0.004). Supplementary Figure S1 provides a heat map of the correlation between each of the clinical and behavioral variables.

**Table 1:** Demographic, behavioral, and clinical characteristics of CD participants and HCs.

	CD (*n* = 25)	HC (*n* = 25)
Age (years)	30 (18–68)	31 (20–65)
BMI (kg/m^2^)	22 (16–33)	26 (18–30)
Male	16	16
Female	9	9
Ethnicity (% Caucasian)	21	22
TNF α (pg/mL)	0 (0–1,233)	9.2 (0–856)
IL-6 (pg/mL)	35.3 (0–259)	12.27 (0–492)
IL1-Beta (pg/mL)	0 (0–1955)	38.5 (0–492)
HADS—Anxiety	5 (1–11)	3.5 (0–15)
HADS—Depression	*3 (0–14)	1(0–8)
Disease duration (years)	7 (1–20)	–
Disease activity defined by Ileocolonoscopy	11	–
Disease activity defined by MRE	12	–
C-reactive protein (mg/dL)	5 (5–224)	–
Faecal calprotectin (μg/g)	458 (18–1800)	–
Harvey Bradshaw index (HBI)	3 (0–9)	–
IBD Fatigue	12 (3–15)	–
Abdominal pain score	10 (0–50)	–
EIM/no EIM	8/17	–

HBI scores (<5 remission, 5–7 = mild disease, 8–16 = moderate disease, >16 = severe disease). IBD fatigue score = (ranges from 0 to 20), where 0 = no fatigue, 1–10 = moderate fatigue, 11–20 = severe fatigue. HAD score (anxiety and depression) 0–7 (normal), 8–10 (mild), 11–14 (moderate), and 15–21 (severe). Twelve patients had evidence of inflammatory disease at MRE, and 8 patients had evidence of inflammatory disease at colonoscopy, 4 had evidence of inflammatory disease as indicated by FCP levels, 1 patient had evidence of inflammatory disease as indicated by CRP levels and *p < 0.005.

### 3.1 Comparison of rs-FC between CD patients and HCs

**Figure 2:**
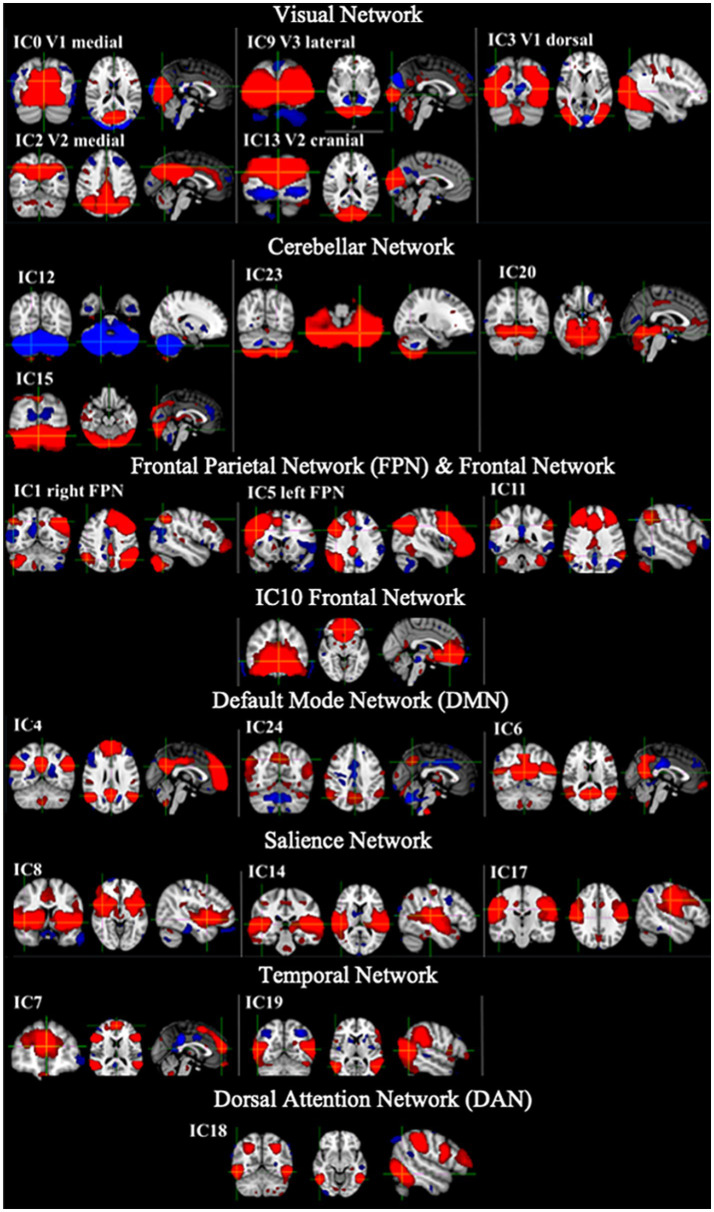
Example RSNs and their associated IC number for the template generated from the HC only which were used in the dual regression analysis. Networks shown include visual networks, cerebellar network, frontal parietal network (FPN), default mode network (DMN), salience network (SN), temporal network and dorsal attention network (DAN). Red = positive networks (2 < z-score < 5) and blue = negative networks (−2 < z-score < −5).

Figure 2 shows example RSNs generated for the template network of the HC group only. This network was used in the dual regression analysis to assess differences in rs-FC between the CD participants and HCs.

Table 2 provides a summary of the regional alterations in rs-FC in RSNs between the CD participants in active disease and HCs. CD participants in active disease showed increased rs-FC compared to HCs in the inferior temporal gyrus and lateral occipital cortex in the dorsal and cranial Visual Network, and in the OFC in the medial Visual Network. Also, greater rs-FC was observed in the FPN in the inferior temporal gyrus, occipital fusiform gyrus, thalamus, caudate, PCC, postcentral gyrus and lingual gyrus in CD participants compared to HCs. Reduced rs-FC in CD participants compared to HCs was shown in the PHG and cerebellum of the DMN, the cerebellum, lingual gyrus and postcentral gyrus of the SN, and the occipital fusiform gyrus and cerebellum in the Cerebellar Network.

**Table 2:** Regional alterations in rs-FC between CD participants and HCs using dual regression analysis and a HC network template.

RSN	L/R	Voxels	Corrected *p*-value	MNI (x, y, z)
CD > HC				
Visual network (IC2&IC3& IC13)				
Inferior temporal gyrus	L	5,585	<0.01	−47, −48, −22
Lateral occipital cortex	L	5,795	<0.01	−38, −68, 19
Lateral occipital cortex	L	7,453	<0.05	−33, −77, 16
OFC	R	5,629	<0.05	22, 25, −17
FPN (IC5&IC11)				
Inferior temporal gyrus	R	5,122	<0.05	46, −40, −28
Occipital fusiform gyrus	L	5,536	<0.01	−25, −72, −14
Thalamus	R	6,299	<0.01	8.6, −6.7, 2.2
Caudate	R	5,544	<0.01	8.6, 16, 0.8
PCC	R	6,619	<0.01	21, −44, 0.1
Postcentral gyrus	R	6,219	<0.01	67, −17, 20
Lingual gyrus	R	5,417	<0.01	25, −50, −8
HC > CD				
DMN (IC24)				
Parahippocampal gyrus (PHG)	L	4,259	<0.05	−23, −32, −24
Cerebellum	L	6,852	<0.05	−18, −50, −30
Salience network SN (IC8)				
Cerebellum	L	6,660	<0.05	−35, −58, −36
Lingual gyrus	R	3,094	<0.05	6, −58, −1.6
Postcentral gyrus	R	5,960	<0.05	60, −13, 27
Cerebellar network (IC12)				
Occipital fusiform gyrus	L	4,315	<0.05	−32, −73, −19
Cerebellum	L	5,990	<0.05	−16, −71, −21

### 3.2 Correlation of pain scores, fatigue scores, disease duration in CD patients

Table 3 shows that in visual and salience RSNs, brain regions negatively correlated with abdominal pain scores in active CD participants, whilst in the frontal network the cerebellum was shown to positively correlate with abdominal pain scores.

**Table 3:** RSNs show significant negative and positive correlations with abdominal pain scores in active CD participants, those which are significant after multiple comparisons are shown in *.

RSN	L/R	Voxels	*P*-value corrected	MNI (x, y, z)
Negative correlation				
Medial visual network V1 (IC0)				
Precuneus	L	4,716	<0.05	−10, −67, 20
Salience network SN (IC8)				
Parietal operculum	R	6,397	<0.05	37, −33, 18
Positive correlation				
Frontal network (IC11)				
Cerebellum	R	5,760	<0.05*	5, −77, −30

Table 4 shows that in the visual network brain regions correlated with disease duration in active CD participants. There was no significant correlation between fatigue scores, or HADS anxiety and depression scores and any of the RSNs.

**Table 4:** RSNs showing significant positive correlations with disease duration in active CD participants.

RSN	L/R	Voxels	*P*-value corrected	MNI (x, y, z)
Positive correlation				
Visual anterior Network (IC2)				
Middle temporal gyrus	R	5,798	<0.05*	−62, −29, −17
Planum temporale	R	5,816	<0.05	50, −30, 14

Those which are significant after multiple comparison are shown in *.

## 4 Discussion

In this work we explore alterations in resting state connectivity in the active phase of CD, which has received limited attention as prior studies generally focus on CD in remission. In this section, alterations in the connectivity patterns seen in the visual network, FPN, DMN, salience and cerebellar networks in active CD relative to HCs are first discussed with relation to CD symptoms and manifestations. Correlations in rs-FC with clinical features of abdominal pain and disease duration are then reviewed, followed by a discussion of study limitations and future research directions.

### 4.1 Alterations in rs-FC in the visual network

Greater rs-FC in the occipital region of the visual network was seen, a finding in line with Kornelsen et al. (2020) who compared a mixed CD group (active/remission, *n* = 7/28) with HCs We also show greater rs-FC in the OFC (an area implicated in executive function), inferior temporal gyrus (visual perception) and lateral occipital (object recognition) in the visual network in active CD participants relative to HCs. Alterations in rs-FC in the visual network may manifest as a result of lower cognitive functioning in CD participants, with impaired information processing speed, task-switching abilities and verbal function having been documented in CD (Golan et al., 2016; Petruo et al., 2017; Nair et al., 2019).

### 4.2 Alterations in rs-FC in the frontal parietal network

A number of rs-FC alterations were evident in the FPN, with greater rs-FC in the FPN in the inferior temporal gyrus (visual perception), occipital fusiform gyrus (object and facial expression recognition), thalamus (relaying and integrating information), caudate (reward), PCC (pain processing), and postcentral gyrus (somatosensory processing) and lingual gyrus (visual information processing) (Table 2). Such changes in the FPN are in agreement with previous studies (Thomann et al., 2017; Kornelsen et al., 2020; Mallio et al., 2020), two of these studies also used an ICA dual regression approach and reported a reduction in rs-FC in the right FPN in multiple frontal, temporal and occipital regions in CD in remission compared with HCs (Mallio et al., 2020), as well as increased rs-FC within the left FPN (in the superior parietal lobe) in CD patients in remission relative to active CD patients (Agostini et al., 2023). Previous neuroimaging studies have shown that the FPN is critical for the regulation of emotions. Our rs-FC findings in the FPN could be implicated in the inhibition of mentalization processes recently highlighted in patients with IBD (Agostini et al., 2019; Engel et al., 2021). Chronic exposure to physical discomfort in the context of CD has been suggested to lead to reduced mentalization (the ability to understand one’s own behavior and the behavior of others) and result in alterations in brain areas that are involved in emotion processing. Altered rs-FC in multiple regions in the FPN, may manifest as heightened sensitivity to visceral sensory information such as increased symptom monitoring, hypervigilance, as well as anxiety around anticipation of abdominal pain, cramps, and diarrhea.

### 4.3 Alterations in rs-FC in the cerebellar network

In the cerebellar network, reduced rs-FC was seen in the occipital fusiform gyrus (object and facial recognition) and cerebellum (motor and emotion) in CD participants compared with HCs. Alterations in rs-FC within the cerebellar network have also been previously reported by Kornelsen et al. (2020) who show greater rs-FC in the cerebellar network in the left superior lateral occipital in CD group (active/remission, *n* = 7/28) compared with HCs. The cerebellar network is associated with action and somesthesis (bodily perception and somatosensory processing) and the rs-FC alteration in CD patients may also be linked with impaired emotional processing, and heightened sensitivity to negative physical and visceral.

### 4.4 Alterations in rs-FC in the DMN

Our finding of alterations in rs-FC in the DMN has been widely reported in CD literature in remission and active disease (Thomann et al., 2017, 2021; Bao et al., 2018; Liu et al., 2018; Hou et al., 2019; Kornelsen et al., 2020; Li et al., 2022; Goodyear et al., 2023). We show reduced rs-FC in the DMN in the parahippocampal gyrus (visuospatial processing and episodic memory) and cerebellum in active CD participants compared with HCs. This may represent disrupted self-referentially processing and self-regulation, which may have implications pertaining to body perception and image as seen in anorexia nervosa (Cowdrey et al., 2014).

### 4.5 Alterations in rs-FC in salience network

In the SN, we identified reduced rs-FC in the cerebellum (motor function and emotion), lingual gyrus (visual information processing) and postcentral gyrus (somatosensory processing) in active CD participants compared with HCs. Alterations in rs-FC in the SN have been documented before (Kornelsen et al., 2020; Prüß et al., 2022), with one study showing greater rs-FC in the SN in the left planum temporale in CD participants in remission compared with HC (Kornelsen et al., 2020). Another study using a ICA dual regression approach showed increased rs-FC in the secondary somatosensory cortex in the SN in active IBD (78% CD, 22% UC) relative to HC (Prüß et al., 2022). The SN has been implicated in orientation toward salient emotional stimuli, conflict monitoring, response choice, information integration, and pain-related processes during acute stimulus-induced pain (Heine et al., 2012). We hypothesize that disrupted connectivity in SN may have implications pertaining to emotional dysregulation and impaired processing of sensory stimuli explaining heightened pain perception, anticipation (Huang et al., 2016; Rubio et al., 2016) and dysregulated psychological stress responses (Agostini et al., 2013).

### 4.6 Association between rs-FC and abdominal pain and disease duration

On correlating with clinical scores, we show that greater abdominal pain was associated with reduced rs-FC in the precuneus (body image and weight consciousness) and the parietal operculum (implicated in pain) and in the medial visual RSN and SN (IC8) respectively. The SN has been implicated in chronic abdominal pain in active CD (Prüß et al., 2022). We further show that greater abdominal pain was associated with greater rs-FC in the cerebellum in the frontal RSN (IC11) (Table 3). Altered rs-FC in the SN, may be linked with heightened reactivity to pain and anticipation of discomfort in CD.

We show that greater disease duration was associated with greater rs-FC in the middle temporal gyrus (language and semantic memory processing) and planum temporale (auditory perception and attention) in the visual anterior RSN (IC2). Altered functional connectivity in regions in the visual network has been reported before (Li et al., 2022), where reduced rs-FC in the left calcarine was associated with greater disease duration. We postulate that disease duration is one of the variables that to an extent affect disease burden which may be related to the changes seen in visual network due to the increased chronic exposure of the CNS to symptom and chronic inflammatory stimuli. We found no significant association between fatigue, anxiety or depression scores and rs-FC in any of RSNs.

### 4.7 Study limitations

There are some limitations of the study. As expected, CD patients were on a number of differing types and dosage of immunosuppressive agents or biological agents as prescribed for their clinical care, which may have influenced their rs-fMRI patterns. A limited number of studies have assessed the effects of immunosuppressive medications on rs-fMRI, which have shown small or minimal effects which are drug dependent. In one study, the effects of anti-TNF therapy and interferon-α treatment was shown to result in small regional patterns of global brain connectivity changes, but the changes did not correlate suggesting independent underlying processes (Martins et al., 2022). In a study of the effects of fingolimod therapy for multiple sclerosis (Bhattacharyya et al., 2020) no changes in motor or FPN rs-fMRI were observed over 24 months. Here, we could not study influence of medications on our rs-fMRI measures, instead we use the CD participants subjective experience of abdominal pain, fatigue at the time of the MRI scan as covariates of interest.

Our ICA approach using a study specific HC network template is a strength as it is a model-free, data driven approach with few priori assumptions, however, it may be less sensitive to inter-individual variation. This study is limited by its sample size and its cross-sectional nature which only provided a small window to assess the association between mild abdominal pain and rs-FC. It is important to note that, due to variations in the data analysis methods, patient population, disease status/activity/severity, sample size/heterogeneity, concomitant medication, and disease-related symptoms, results need to be interpreted with caution when contrasting findings with other published literature and so this warrants further investigation in a large homogenous sample of active CD.

### 4.8 Future research directions

Future studies assessing rs-FC in CD, could use a non-gastrointestinal chronic inflammatory disease patient group as a comparator to dissect the relationship between gastrointestinal, systemic inflammation and rs-FC. In future, it would also be of interest to perform studies of rs-FC whilst longitudinally tracking CD patients during the active phase of the disease and in remission, to see if the change in connectivity in differing network patterns (visual, FPN, cerebellar, SN, DMN) are associated with improved disease outcomes and cognitive function. This could then be related to underlying changes in symptoms and manifestations. Identification of a neural phenotype through rs-FC specific to active CD status, may help guide intervention constituting a neurobehavioral precision medicine approach to treatment.

## 5 Conclusion

Alterations in rs-FC between the CD and HC groups in the RSNs implicated in cognition, attention, emotion, and pain may represent neural correlates of chronic systemic inflammation, abdominal pain, and disease duration constituting a unique neurobehavioral phenotype specific to active CD.

## Data availability statement

The raw data supporting the conclusions of this article will be made available by the authors, without undue reservation.

## Ethics statement

The studies involving humans were approved by the National Research Ethics Service (NRES) Committee East Midlands (REC reference 14/EM/0192 on 10/07/2015) and the protocol was registered with clinicaltrials.gov (NCT02772458). The studies were conducted in accordance with the local legislation and institutional requirements. The participants provided their written informed consent to participate in this study.

## Author contributions

GT: Investigation, Methodology, Analysis, Writing – review & editing. SE: Data curation, Supervision, Writing – review & editing. SR: Data curation, Writing – review & editing. SF: Conceptualization, Funding acquisition, Investigation, Methodology, Supervision, Writing – review & editing. GM: Conceptualization, Funding acquisition, Investigation, Supervision, Writing – review & editing.

## Footnotes

^1^ http://fsl.fmrib.ox.ac.uk/fsl/fslwiki/
