# Supplementary material for: An examination of resting-state functional connectivity in patients with active Crohn’s disease
Source: Front Neurosci. 2023 Dec 6;17:1265815. doi: 10.3389/fnins.2023.1265815 (PMC10731262; doi:10.3389/fnins.2023.1265815)
Supplement: Supplementary file 1 [file Table_1.DOCX]

**Supplementary Material**

1. **Introduction**

The most commonly reported RSNs in the literature are shown with the brain regions involved and behavioural interpretations of the different RSNs shown in Table s1

| RSN | Regions | Function |
| --- | --- | --- |
| Lateral visual | Middle temporal visual association area at the temporal-occipital junction | Most important in complex (emotional) stimuli (e.g. viewing faces/films) |
| Medial visual |  | Simple visual stimuli (e.g. flickering checkerboard) |
| Occipital visual |  | High order visual stimuli (orthography)(1) |
|  |  |  |
| Sensorimotor network | Involves SMA/MCC, bilateral primary motor cortex, and bilateral middle frontal gyri | The sensorimotor network resembles the activations seen in motor and sensory tasks(2) |
| Dorsal attention network (DAN) | Involves the intraparietal sulcus, the junction of the precentral and superior frontal sulcus (frontal eyelids) | Orienting focus to a particular task(3), also involved in cognitive top-down processing, which involves factors such as knowledge, expectation and current goals(3) |
| Salience network | Involves fronto-insular and ACC with connections to the subcortical and limbic region | Implicated in orientation toward salient emotional stimuli, conflict monitoring, response choice, information integration, pain-related processes during acute stimulus-induced pain(4) |
| Temporal RSN | Involves inferior frontal gyrus, medial temporal gyrus, superior temporal gyrus | Involved in reading and language processing (5) |
| DMN | Involves precuneus, PCC, mesiofrontal/ACC and temporoparietal junction regions | Less active during task performance, compared with resting state, linked to self-related and internal process such as thoughts independent of any stimulus(6) |
| FPN | Involves frontal and parietal lobules and cingulate gyrus | FPN has been associated with different functions, i.e., executive function working memory, language, attention and visual processes(5). |
| Cerebellar network | Cerebellum | Associated with action and somesthesis (sense of touch, pressure, temperature, etc.)(1) |
| Executive control network (ECN) | Involves the medial frontal gyrus, superior frontal gyrus, and ACC | Involved in tasks relying on executive functions, such as control processes and working memory. (7). |

Table s1. Different RSNs and their behavioural interpretation

1. **Results**

| **ID** | **Disease phase** | **Sex** | **Age (years)** | **BMI (kg/m^2^)** | **Montreal** | **Smoking** | **Medication** | **MRE** | **Colonoscopy** | **disease duration** | **CRP** | **FCP** | **HBI** | **EIM** |
| --- | --- | --- | --- | --- | --- | --- | --- | --- | --- | --- | --- | --- | --- | --- |
| 1 | A | M | 30 | 28 | A1L1B3 | Never | TH | Evidence of inflammatory disease |  | 15 | 5 | 30 | 8 |  |
| 2 | A | M | 20 | 19 | A2L1B1 | Never | AZA, |  |  | 3 | 5 | 1027 | 4 |  |
| 3 | A | M | 22 | 24 | A2L1B1 | Never | AZA, | Evidence of inflammatory disease |  | 5 |  |  | 3 |  |
| 4 | A | M | 30 | 29 | A1L1B3 | Never | TH ,INF, |  | Evidence of inflammatory disease | 17 |  |  | 5 | aphthous ulcers |
| 5 | A | M | 44 | 33 | A2L1B2 | Unknown | INF, aminosalicylates | Evidence of inflammatory disease |  | 9 |  |  | 2 | Arthralgia |
| 6 | A | M | 32 | 30 | A2L1PB2 | Unknown | TH, AD | Evidence of inflammatory disease |  | 14 |  | 574 | 8 | Arthralgia, erythema nodosum |
| 7 | A | M | 63 | 27 | A2L3B1 | Ex-smoker |  |  | Evidence of inflammatory disease | 20 | 5 | 167 | 3 |  |
| 8 | A | F | 20 | 16 | A2L3B2 | Unknown | NIL |  | Evidence of inflammatory disease | 1 |  |  | 5 | Arthralgia, aphthous ulcers |
| 9 | A | M | 28 | 19 | A2L1B1 | Unknown | Tacrolimus, |  | Evidence of inflammatory disease | 1 | 5 | 174 | 3 |  |
| 10 | A | M | 38 | 31 | A2L1B1 | Ex-smoker | AD, TH |  |  | 7 | 6 | 785 | 8 | uveitis |
| 11 | A | F | 67 | 22 | A3L3B1 | Ex-smoker | MTX, | Evidence of inflammatory disease |  | 1 | 224 | 1800 | 8 | aphthous ulcers, abscess |
| 12 | A | F | 38 | 27 | A2L3PB2 | Never | TH | Evidence of inflammatory disease | Evidence of inflammatory disease | 18 | 10 |  | 2 | Abscess |
| 13 | A | M | 68 | 19 | A3L3B1 | Ex-smoker |  |  | Evidence of inflammatory disease. | 1 | 5 | 458 | 9 |  |
| 14 | A | M | 25 | 22 | A1L3B1 | Never | AD | Evidence of inflammatory disease | Evidence of inflammatory disease | 9 | 5 | 607 | 1 |  |
| 15 | A | F | 20 | 20 | A1L3PB1 | Never | INF |  |  | 8 | 25 |  | 0 |  |
| 16 | A | M | 18 | 22 | A1L2B1 | Unknown | TH |  |  | 6 | 5 | 1266 | 8 |  |
| 17 | A | M | 41 | 22 | A2L1B1 | Current | MTX, |  |  | 2 | 5 | 414 | 5 |  |
| 18 | A | F | 19 | 22 | A2L3B3P | Never | NIL |  | Evidence of inflammatory disease | 1 | 11 | 1800 | 1 |  |
| 19 | A | M | 25 | 30 | A2L3B3P | Never | TH | Evidence of inflammatory disease |  | 8 | 25 | 1800 | 2 |  |
| 20 | A | F | 24 | 22 | A1L2B1 | Never | AD |  | Evidence of inflammatory disease | 11 |  |  | 1 |  |
| 21 | A | F | 62 | 20 | A3L3B1 | Ex-smoker | AD, TH, | Evidence of inflammatory disease |  | 2 | 5 | 404 | 1 |  |
| 22 | A | F | 32 | 29 | A2L3B1 | Never | TH | Evidence of inflammatory disease | Evidence of inflammatory disease | 10 |  |  | 6 | Arthralgia |
| 23 | A | F | 31 | 21 | A2L3PB1 | Unknown | Nil | Evidence of inflammatory disease |  | 10 | 5 | 194 | 6 |  |
| 24 | A | M | 41 | 19 | A2L2B2 | Never | AD, TH |  | Evidence of inflammatory disease | 7 | 5 | 434 | 1 |  |
| 25 | A | M | 18 | 23 |  |  | TH, Inf | Evidence of inflammatory disease |  | 1 | 27 | 18 | 0 |  |

Table s2 : Patient characteristics

A= ACTIVE DISEASE, M = MALE , F= FEMALE, EIM = EXTRAINTESTINAL MANIFESTATIONS, AD=ADALIMUMAB, AZA= AZATHIOPRINE INF=INFLIXIMAB, , MTX=METHOTREXATE, TH = THIOPURINE Montreal classification Age (A); A2=17-40 years, A3= over 40 years. Disease location (L); L1 terminal ileum, L2= colon, L3= ileocolon, L4= upper gastrointestinal tract (a perianal disease modifier may be added in the presence of perianal fistulas). Disease behavior (B); B1= non-stricturing non-penetration, B2= structuring, B3 penetrating


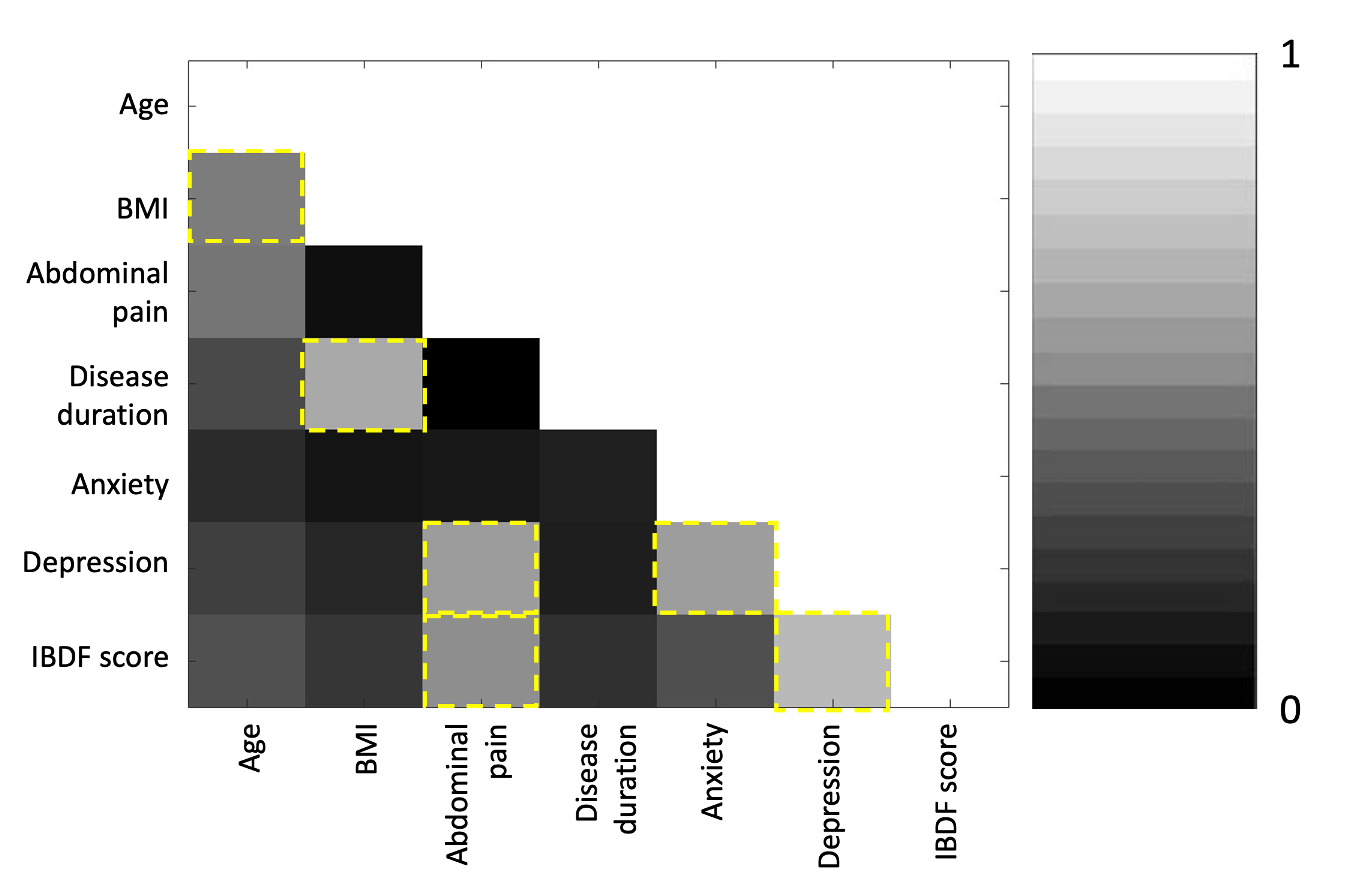


Figure s1. A heat map of the correlation between each of the clinical and behavioural variables. Yellow squares indicate a significant correlation.
